# Supplementary material for: Assessing the utility of CASP14 models for molecular replacement
Source: Proteins. 2021 Aug 21;89(12):1752–69. doi: 10.1002/prot.26214 (PMC8881082; doi:10.1002/prot.26214)
Supplement: Supplementary file 1 — APPENDIX S1: Supporting information [file PROT-89-1752-s001.docx]

# Supplementary Material for:

# Assessing the utility of CASP14 models for molecular replacement

Authors: Claudia Millán^1^*, Ronan M. Keegan^2^*, Joana Pereira^3§^, Massimo D. Sammito^1^, Adam J. Simpkin^4^, Airlie J. McCoy^1^, Andrei N. Lupas^3^, Marcus D. Hartmann^3^, Daniel J. Rigden^4^, Randy J. Read^1^

Affiliations:  ^1^ Department of Haematology, University of Cambridge, Cambridge Institute for Medical Research, Cambridge CB2 0XY, United Kingdom
^2^ Scientific Computing Dept., Science and Technologies Facilities Council, UK Research and Innovation, Didcot, Oxfordshire, United Kingdom

^3^ Max Planck Institute for Developmental Biology, Max-Planck-Ring 5, Tübingen, Germany
^4^ Institute of Systems, Molecular and Integrative Biology, Biosciences Building, Crown Street, Liverpool L69 7BE, United Kingdom


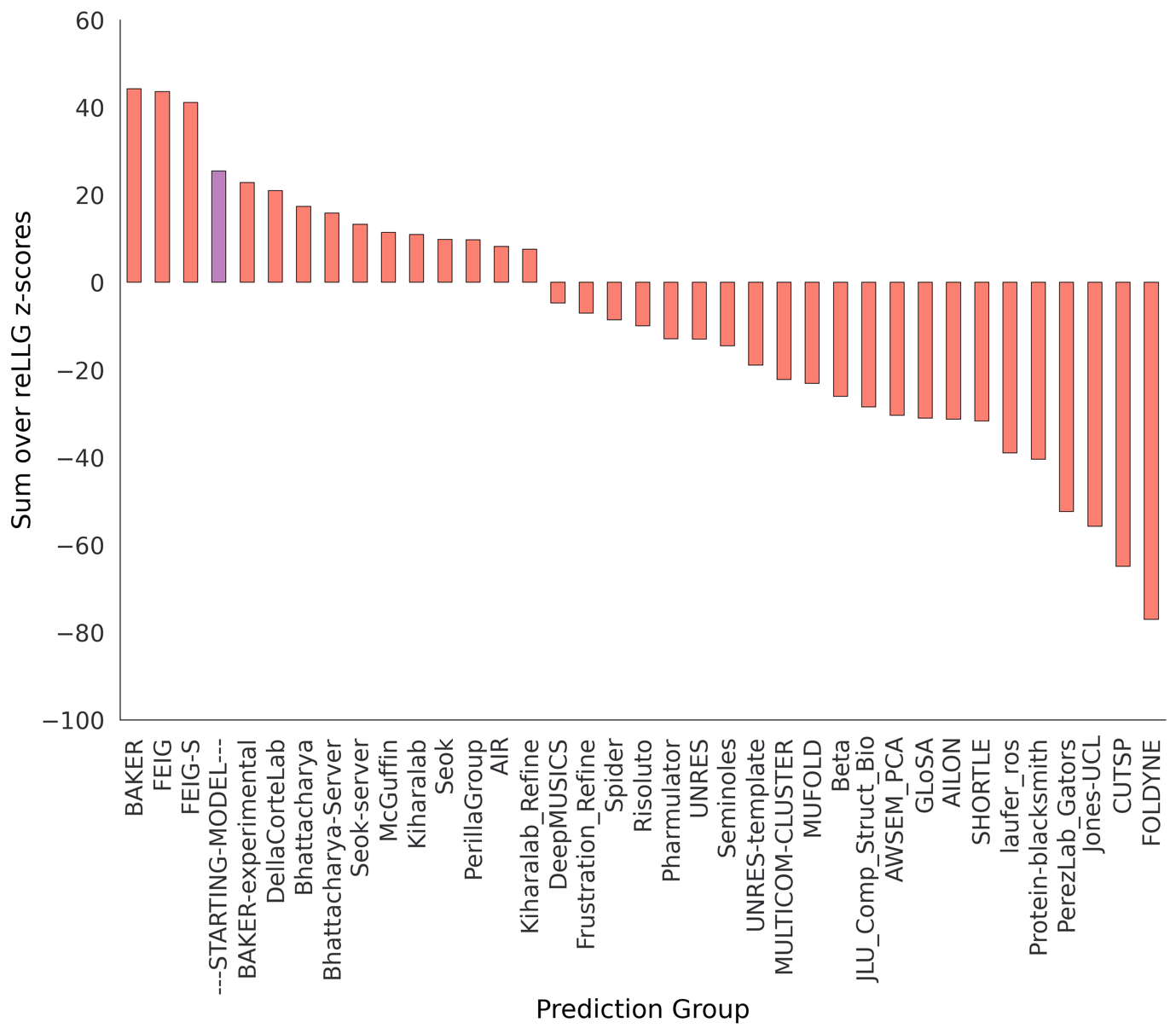


Figure S1. Alternative ranking of refinement groups by reLLG Z-score computed with constant B-factors. By this ranking, which focuses only on coordinate accuracy, only 3 groups outperform the starting model, which was also scored using constant B-factors.


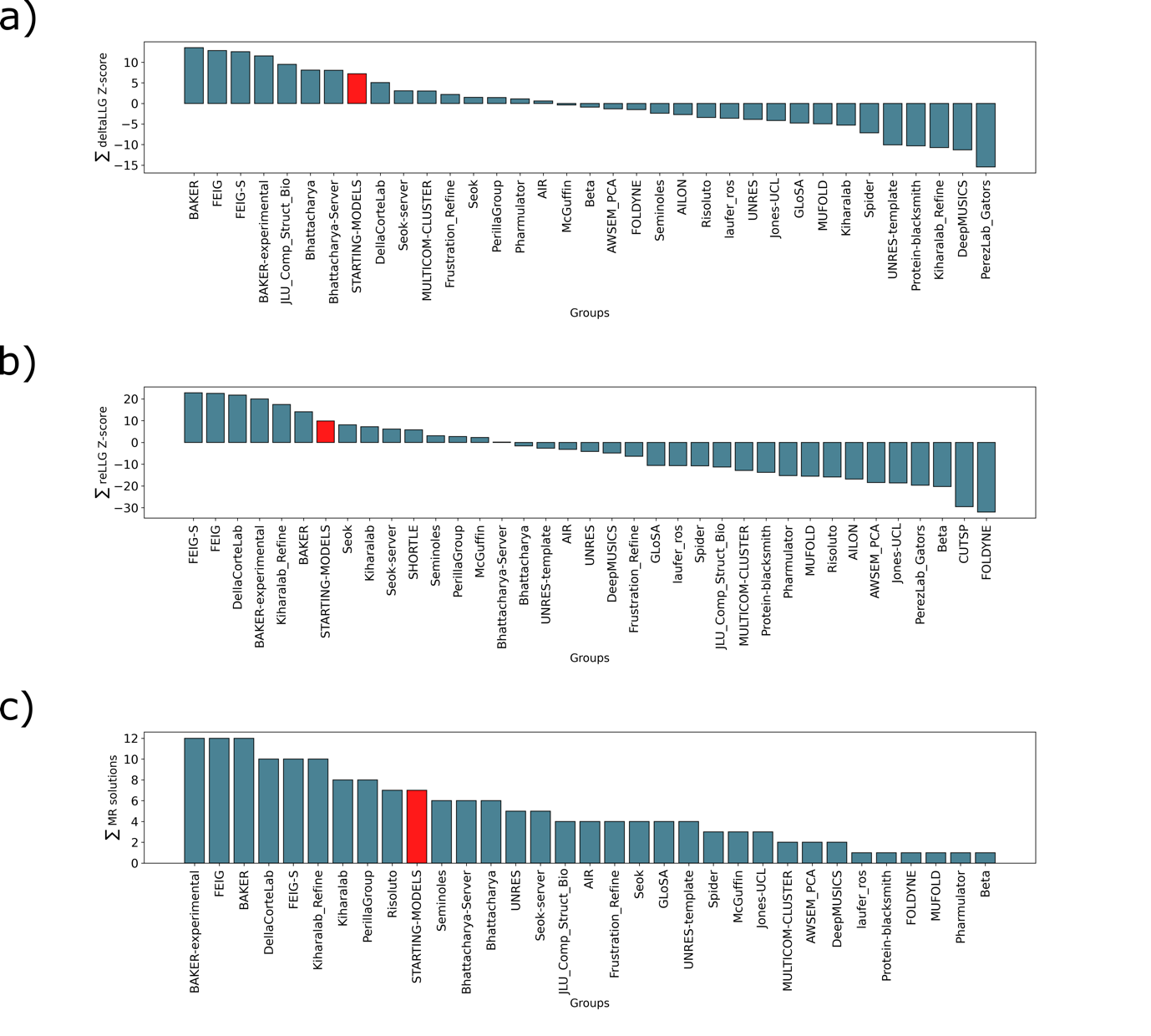


Figure S2. A comparison of alternative ranking strategies for the refinement methods against the 20 targets assessed with MR. These are ranked on the sum of dLLG Z-scores (a), the sum of reLLG Z-scores (b) and the sum of total solutions in MR (c). The scores were calculated using only model 1 and the naïve predictor is shown in red.
